# Post COVID-19 pulmonary complications; a single center experience

**DOI:** 10.1016/j.amsu.2021.103052

**Published:** 2021-11-10

**Authors:** Okba F. Ahmed, Fahmi H. kakamad, Bnar J. Hama Amin, Berwn A. Abdullah, Marwan N. Hassan, Rawezh Q. Salih, Shvan H. Mohammed, Snur Othman, Gasha S. Ahmed, Abdulwahid M. Salih

**Affiliations:** a Mosul Cardiac Center, Mosul, Iraq; b Department of Surgery, College of Medicine, University of Sulaimani, Sulaimani, Kurdistan Region, Iraq; c Smart Health Tower, Madam Mitterrand Street, Sulaimani, Kurdistan, Iraq; d Kscien Organization for Scientific Research, Hamdi Street, Al Sulaymaniyah, Kurdistan Region, Iraq; e College of Health Sciences, Medical Laboratory Science Department, University of Human Development, Sulaimani, Kurdistan, Iraq; f Department of Medical Microbiology, Faculty of Medicine, Gaziosmanpasa University, Tokat, Turkey

**Keywords:** COVID-19, SARS-CoV-2, Pulmonary, Complications, Post COVID-19 syndrome

## Abstract

**Introduction:**

Although the rate and severity of complications after coronavirus 2019 disease (COVID-19) resolution is currently unknown, evidence regarding their presence is increasing in the literature. This study presents a series of cases with post COVID-19 short-term pulmonary complications.

**Methods:**

This is a single center retrospective case series study. The demographic and clinical data were collected from the center's electronic records. All the included cases were confirmed COVID-19 patients who had pulmonary complications even after their recovery.

**Results:**

Nineteen COVID-19 patients were involved in this study. Fourteen of them were male (73.7%) and only 5 (26.3%) cases were female, with a mean age of 52.05 years (26–77). All of the patients developed severe COVID-19 and were admitted to intensive care unit (ICU). The average infection duration was 13.5 days (10–21). The most common complaints after recovery from COVID-19 were shortness of breath, fever, and hemoptysis. Computed tomography scan showed different pulmonary abnormalities between the cases. Different surgical procedures were performed for the patients according to their conditions, such as decortications, lobectomy, and bullectomy. More than half of the patients (n = 10) recovered and were discharged from hospital without complications, five patients were admitted to the ICU, 3 cases developed mucormycosis, and one case passed away.

**Conclusion:**

Following the resolution of COVID-19, patients may experience severe pulmonary complications that may last for months and can affect quality of life, ICU admission, or even death.

## Highlights

• Coronavirus 2019 disease (COVID-19) is not only a respiratory syndrome.
• COVID-19 patients may suffer from complications even after its resolution.
• The rate and severity of long-term pulmonary complications of COVID-19 is unknown.
• This study presents a series of cases with post COVID-19 short-term pulmonary complications.

## 1 Introduction

Corona Virus Disease-19 (COVID-19) is not only a respiratory syndrome, the produced level of inflammatory substances produced in response to the virus can lead to the destruction of the target tissue and can even go beyond body barriers of innate immunity, reaching other organs through hematogenous spread [1]. In addition, ARDS can induce irreversible scarring of the lung tissue, which can lead to long-term respiratory insufficiency [2]. Even though active COVID-19 is well known to be associated with pneumonia and a wide range of systemic diseases, evidence regarding complications after COVID-19 resolution is increasing in the literature [1,3]. The rate and severity of the long-term pulmonary complications of COVID-19 is presently unknown; however, recent studies have reported various persistent respiratory symptoms months after recovery from SARS-CoV-2 infection [4].

The current study aims to present a series of cases whom have developed short term pulmonary complications after recovering from COVID-19.

## 2 Methods

### 2.1 Registration

In accordance with Helsinki declaration, the current study has been registered - “Every research study involving human subjects must be registered in a publicly accessible database before recruitment of the first subject”. The study was recorded in the Chinese Clinical Trial Registry, with a registration number of: ChiCTR2100051569, the link is https://www.chictr.org.cn/hvshowproject.aspx?id=101316.

### 2.2 Study design

This study is a single center retrospective case series. The paper was written in accordance with PROCESS 2020 guidelines [5].

### 2.3 Setting

Patients were collected in one of the governmental hospitals in Iraq throughout a period of 7 months; from January 2021 until July 2021. Ethical and scientific approval was acquired from Al-Jamhori teaching hospital.

### 2.4 Data collection and analysis

Data were collected from the center's database. Microsoft excel 2019 was used for collection of the data. Statistical Package for the Social Sciences (SPSS) Version 25 was used for coding of the data and conducting Data analysis.

### 2.5 Inclusion and exclusion criteria

Those patients were included who were infected with SARS-CoV-2 and developed pulmonary symptoms in less than 6 months of recovery, or already had the symptoms during active infection but persisted even after recovery. These symptoms should be clearly related to COVID-19 infection. Known cases of lung cancer, tuberculosis, asthma, chronic obstructive pulmonary disease, pulmonary fibrosis, and other chronic lung diseases were excluded from the study.

Diagnosis of COVID-19 was confirmed through real-time reverse transcriptase polymerase chain reaction (RT-PCR) with nasopharyngeal swab or oropharyngeal swab. Other accepted ways of diagnosis were: viral PCR, IgG and IgM, and chest computed tomography (CT) scan with obvious sign and symptoms of COVID-19.

## 3 Results

A total of 19 patients infected with SARS-CoV2 were involved in this study. The majority of the cases were male (73.7%, n = 14), and only 5 cases (26.3%) were female. The mean age was 52.05 years, ranging from 26 to 77 years. Fourteen (73.7%) patients were nonsmokers. Diabetes was the most common comorbidity. All of the patients developed severe COVID-19 and were admitted to intensive care unit (ICU). The duration of the infection varied among the individuals, ranging from 10 to 21 days with a mean of 13.5 days. Patient recovery from COVID-19 was confirmed via RT-PCR. The most common complaint by the patients after their recovery from the infection were shortness of breath, fever, and hemoptysis. All of the patients were sent for CT scan which showed different pulmonary abnormalities and findings between the cases (Table 1). Different surgical procedures were performed for the patients according to their conditions, such as decortications, lobectomy, and bullectomy, all of which were performed through thoracotomy, except for one instance which was done by Clamshell incision. More than half of the patients (n = 10) recovered and were discharged from hospital without complications, five patients were admitted to the ICU, 3 cases developed mucormycosis, and one case passed away. Among patients with mechanical ventilation, three of them were diabetics (60%, compared to others 14% had diabetics, p-value ˂0.001).

**Table 1:** Demographic and clinical characteristics of participants.

Variable	N (%)
Sex	
Male	14 (73.7)
Female	5 (26.3)
Smoker	5 (26.3)
Yes	14 (73.7)
No	
Past medical history	
DM	8 (42.1)
IHD	1 (5)
TB	1 (5)
Negative	10 (52.6)
Admitted to ICU due to COVID-19	19 (100)
Yes	0
No	
Mechanical ventilation	
Yes	5 (26.3)
No	14 (73.7)
Chief complaint after recovery from COVID-19	
SOB	14 (73.7)
Fever	2 (10.5)
Cough	1 (5)
Hemoptysis	2 (10.5)
Post COVID-19 chest CT findings	
Empyema	10 (52.6)
Air space	5 (26.3)
Cavitary lesion	3 (15.8)
Bronchiectasis	1 (5)
Procedure	
Decortication	13 (68.4)
Lobectomy	4 (21)
Bullectomy	2 (10.5)
Follow up	
Recovered	10 (52.6)
ICU admission	5 (26.3)
Mucormycosis	3 (15.8)
Death	1 (5)

ICU: intensive care unit. COVID-19: corona virus disease-2019. CT: computed tomography scan. DM: diabetes mellitus. IHD: ischemic heart disease. TB: tuberculosis.

## 4 Discussion

Although the majority of SARS-CoV-2 infected cases recover completely, for a considerable proportion of patients surviving COVID-19 may be the beginning of many battles on the long way to full recovery, as they have long-term morbidities that vary in severity; ranging from moderate to devastating complications [6]. Post COVID-19 syndrome is the term denoted to this complex condition which includes multi-organ consequences after the infection has subsided. It includes everything from physical and cognitive impairments to functional limitations and exercise impairment, all of which contribute to a decreased quality of life [7].

Though there is not enough evidence to define and identify post-COVID-19 conditions conclusively, however, emerging data as well as prior experiences with other serious respiratory illnesses can be used to predict long-term consequences [8]. Amongst COVID-19 survivors, a wide range of pulmonary symptoms including dyspnea on exertion, restrictive pulmonary physiology, decreased diffusion capacity, as well as fibrotic lung lesions have been documented, all of which have been linked to the severity of the acute illness [9]. In a study of healthcare providers with mild COVID-19, 26% developed moderate to severe symptoms for 2 months and 15% for 8 months [10]. Following severe COVID-19, a significant number of cases will be at risk for long-term problems. Because of the high frequency of respiratory failure and the necessity for mechanical ventilation in cases of severe clinical symptoms, there have been growing concerns regarding pulmonary sequelae, particularly pulmonary fibrosis (PF) [11]. Long-term pulmonary illness in COVID-19 survivors is currently little understood, but it is becoming a key concern for the medical community [12].

It has been found that SARS-CoV-2 attaches to the respiratory epithelium and attacks the alveolar cells by entering through angiotensin converting enzyme 2 (ACE2) receptors, this in combination with cytokine storm makes the alveoli vulnerable to rupture, resulting in air leakage and the development of cystic air space lesions, and cause lung cystic abnormalities in up to 10% of the patients [13,14]. When SARS-CoV-2 infection starts, pleural effusion can occur with varying incidence. The pleural fluid contains more proteins and white blood cells which are mostly neutrophils. Bacteria may infiltrate the pleural area, causing fibrin deposition and loculation development, culminating in empyema. Patients may be asymptomatic at the time of presentation due to the slow respiratory compensation of this process [15]. Bacterial super infection and subsequent development of pleural empyema may have been promoted by the combination of acute inflammatory state with the onset of reactive pleural effusion [16].

In COVID-19 patients, bilateral pneumonia is a common clinical manifestation, and in certain cases with complicated respiratory pathophysiology, such as pneumothorax, pneumomediastinum, and empyema, have been documented. To our knowledge, five cases of empyema caused by COVID-19 have been recorded, including two cases of pleural fistula. All five patients underwent surgical interventions, such as decortication, and four of them had a favorable clinical outcome [17]. Empyema has long been thought to be a surgical condition, with open decortication being the most effective therapy [18]. However, because of the small number of cases and the procedure's risk grade, surgical treatment of pleural empyema in COVID-19 patients is still to be described in the literature. Pleural empyema is a rare but possible complication, it is a life-treating disease, which can worsen the COVID-19 disease, and it should be treated immediately to improve patient's status and chance of recovery [16]. Most of the patients in the current study with empyema were managed through decortication, except for one case who underwent lobectomy.

It is reported that SARS-CoV-2 pulmonary lesions have a predominantly peripheral and subpleural distribution, and they might be associated with the presence of pneumatocele which is a thin-walled cystic lesion that is typically linked to acute pneumonia and disappears on its own. Pneumothorax can result from a ruptured pneumatocele, therefore cautious monitoring is necessary. Although the origin and method of pneumatocele formation in COVID19 remains unknown, widespread alveolar damage caused by SARS-CoV-2 infection followed by airway wall necrosis can result in pneumatocele [19]. In a study by Martinelli and associates, the biggest series of pneumothoraxes involving nonventilated patients was documented, which shed light on the relationship between pneumothorax and COVID-19. According to their findings, the complication of pneumothorax is more common in men (3.3:1) [20].

The treatment of pneumothorax in ARDS patients who are being invasively ventilated might be difficult. Previous reports have described intubated COVID-19 patients developing pneumothorax that was resistant to chest drain placement and eventually needed surgery [21]. Martinelli et al. reported a case of COVID-19 related pneumothorax, the air leak was persistent despite two chest drain insertions and definitive management was achieved only after bullectomy and pleurodesis [20]. Surgical treatment can be beneficial for certain patients, this is in line with the British Thoracic Society's recommendation that persistent pneumothorax and air leak should warrant surgical referral [22]. In the current study bullectomy was done for air cyst (post-operative histopathology confirmed pneumatocele), and four patients underwent lobectomy (one for bronchiectasis, two for cavitary lesion, and one for empyema).

There are crucial limitations for this study, the sample size is small, we have few comparison groups, and the follow up period short.

In conclusion, following the resolution of COVID-19, patients may experience severe pulmonary complications that could last for months and can lead to a decreased quality of life, ICU admission, or even death. This study highlighted the need for further long-term clinical follow-up of COVID-19 patients.

## Provenance and peer review

Not commissioned, externally peer-reviewed.

## Ethical approval

Approval was taken from Kscien organization for scientific research.

## Sources of funding for your research

No source to be stated.

## Author contribution

Okba F. Ahmed: thoracic surgeon managing the cases, following up the patient, and final approval of the manuscript.

Fahmi H.kakamad, Bnar J. Amin, Berwn A. Abdullah, Marwan N. Hassan, Rawezh Q.Salih, Shvan H.Mohammed, Snur Othman, Gasha S. Ahmed, Abdulwahid M. Salih: literature review, writing the manuscript, final approval of the manuscript.

## Consent

Consent has been taken from the patient and the family of the patient.

## Registration of research studies

The study was recorded in the Chinese Clinical Trial Registry, with a registration number of: ChiCTR2100051569, the is link is https://www.chictr.org.cn/hvshowproject.aspx?id=101316.

## Guarantor

Fahmi Hussein Kakamad is the Guarantor of submission.

## Declaration of competing interest

There is no conflict to be declared.
